# Metabolic dysfunction-associated steatotic liver disease (MASLD) is under-recognised: Should hepatic elastography be integrated into early risk stratification for people with type 2 diabetes?

**DOI:** 10.1016/j.clinme.2026.100629

**Published:** 2026-07-20

**Authors:** Aftab Ala, Ponnusamy Saravanan

**Affiliations:** The Roger Williams Institute of Liver Studies, King’s College London, London, UK; King’s College Hospital NHS Foundation Trust, Denmark Hill, London, UK; Warwick Medical School, Warwick Applied Health, University of Warwick, Coventry, UK; Centre for Global Health, University of Warwick, Coventry, UK; Centre for Early Life, University of Warwick, Coventry, UK; Diabetes, Endocrinology and Metabolism, George Eliot Hospital NHS Trust, Nuneaton, UK

Metabolic dysfunction-associated steatotic liver disease (MASLD), particularly its progressive form metabolic associated steatotic hepatitis (MASH), has become the second leading indication for liver transplantation in the United Kingdom, surpassed only by alcohol-related liver disease, reflecting the growing burden of metabolic liver disease and its complications.^1^ Alarmingly, the escalating prevalence of childhood obesity is driving a parallel epidemiological shift, with hepatic steatosis and type 2 diabetes (T2D) emerging at increasingly younger ages, raising concerns about a longer lifetime exposure to cardiometabolic and liver-related complications.^2^ Of particular concern, the rising burden of MASLD is evident across both high-income and low- and middle-income countries, emphasising that this is no longer a disease confined to affluent societies but a global health challenge with substantial implications for liver-related morbidity, mortality and healthcare expenditure.^3^ As with any specialties, the focus of liver care is increasingly moving towards prevention rather than treatment, recognising that early identification and intervention offer the greatest opportunity to reduce the burden of advanced liver disease, liver cancer and liver-related mortality.

Despite MASLD, in all its different names, being a well-known entity for a considerable time, it still does not get adequate priority for proactive screening and diagnosis even in people living with T2D. The same focus on identification of patients and at-risk groups given to viral hepatitis and alcohol-related liver disease has not been replicated with MASLD. The global prevalence of MASLD in T2D is estimated to be up to 69%, with predilection particularly to North African, Middle Eastern, Latin American and South Asian populations.^4^ The overlap includes obesity (though not always), genetic predisposition and visceral adiposity. The hepatic consequences and natural history of MASLD is its progression to MASH and then to cirrhosis. Cirrhosis can occur in 25% of patients with MASH and typically asymptomatic until decompensation occurs with encephalopathy and features of portal hypertension, including variceal bleeding and ascites. Hepatocellular carcinoma can occur with or without cirrhosis in the presence of MASLD and T2D are more likely to develop primary hepatocellular carcinoma (HCC), with five-year mortality of 74.6%.^5^

In addition to the higher risk of liver disorders, MASLD is a multisystem disease and increases the future risk of ischaemic heart disease, chronic kidney disease and osteoporosis.^6^, ^7^ Lean MASLD represents 20% of the worldwide prevalence but is much more common in South and East Asian populations.^8^ This sub-group also carries similar hepatic and cardiometabolic risks. Thus, proactive screening, diagnosis and further risk stratification of MASLD is warranted to potentially employ targeted strategies to reduce these future complications.

Liver biopsy is typically required to diagnose MASH and assess the severity of fibrosis. FIB-4 score/index was developed as a non-invasive tool (NIT) for risk stratification in primary care to reduce the referral burden to hepatologists. However, FIB-4 is less reliable in a younger population (<35 years old – less validated) and false positives can occur in T2D and older age groups due to altered serum ALT and platelet profiles. Vibration-controlled transient elastography (VCTE), such as FibroScan, was developed as a NIT for assessing liver fibrosis.^9^

Current guidelines for NIT recommend a two-step approach for the stratification of MASLD starting with FIB-4 and followed by VCTE or alternate test. NICE and EASL-EASD-EASO guidelines suggest individuals with intermediate or high non-invasive FIB-4 scores should undergo further assessment.^10^, ^11^ We propose screening should be undertaken annually in T2D and once diagnosed with MASLD testing should be undertaken for advanced fibrosis every three years. For those at higher risk of developing HCC, annual FIB-4 score assessment with six-monthly surveillances would be a sensible approach. Due to the limitations of FIB-4 score-based screening in certain populations, others have explored the utility of liver stiffness (LSM) assessment in patients with T2D. In 4,781 participants, Chen *et al*.^12^ applied a liver stiffness (LSM) threshold of 10 and 15 kPa for further risk stratification and future hepatic elastography. This approach may offer further risk stratification for those with high FIB-4 scores, although more work is needed to validate these thresholds.

In this context, work by Tinoco *et al*. in this issue is interesting as it offers one such alternative detection/risk stratification pathway solution for a Mexican population with T2D in their primary care health system.^13^ They assessed the feasibility and evaluation of a stepwise diagnostic pathway for identifying the multiple facets of MASLD in 454 people with T2D. Their pathway identified 51.5% with MASLD and 22.2% with MASH. Through stepwise assessment, 22.7% showed intermediate-to-high fibrosis risk, and among those who underwent FibroScan, advanced fibrosis was confirmed in 27.9% of individuals with increased risk, yielding an estimated prevalence of 10.6% in the MASLD group and 5.9% overall. In multivariable analysis, female sex, elevated liver enzymes, and higher body mass index were independently associated with MASLD, with a non-linear association across BMI categories.

It is acknowledged that MASLD remains unrecognised in diabetes care due to the significant lack of robust structured detection pathways in primary healthcare. Strengthening primary care capacity remains crucial for seamless systematic integration of MASLD identification in routine diabetes care and diabetes care bundles. Such strengthening is recognised to identify patients where early intervention can be achieved to prevent progression and surveillance can be offered. While Tinoco *et al*.^13^ show that this approach in Mexican primary care system is feasible, it requires replication in other settings, both within Mexico and beyond. The wider adoption beyond hepatology clinics to primary and secondary care is indeed poor worldwide and further work is needed on best approaches in different healthcare settings and populations. Critical questions, such as ‘does routine FibroScan based fibrosis assessment and management of MASLD in adults with T2D improve cardiovascular and liver related outcomes?’ need to answered. In the era of the rapidly expanding role of GLP-1, GIP and other peptide therapies for weight loss and glycaemic control, clarification on their role in reducing rates of MASLD and MASH and future risk of cirrhosis, HCC and cardiometabolic disorders is urgently needed.^14^

We believe early identification and risk stratification of liver disease within diabetes care pathways may therefore offer substantial clinical and economic benefits through the prevention of liver failure and hepatocellular carcinoma; however, the magnitude of this impact remains insufficiently explored and merits prospective evaluation. One notable contribution in this area is a recent evaluation of the potential economic impact of liver disease interventions using generalised cost-effectiveness analysis (GCEA) across 12 countries, spanning all six WHO regions, including Brazil, Chile, Germany, Japan, Italy, Saudi Arabia, South Africa, Spain, Sweden, Tanzania, Thailand, and the United States.^15^ Specifically, the cohort transition model simulated liver disease development and its impact on a synthetic cohort aged 19 years and older over their life span across 10 liver health states. In this economic evaluation modelling study, screening followed by ILS was found to be cost effective management for MASH and liver fibrosis among people living with TD2 in all countries. These results can help inform future health policy decision making and further the development of WHO recommended interventions across the NCO.

The interpretation of aetiology specific FibroScan scores in various sub-populations, e.g., metabolic alcohol related liver disease (MET-ALD), viral hepatitis, hepatitis C, alcohol related liver disease requires validation to ensure we follow evidence-based approaches. In addition, significant exploitation of AI based interpretation to reduce operator dependency and variability as well as the need for specialist expertise could offer additional solutions to implement the screening approaches. Risk stratification and identifying high-risk patients early in their natural history would offer targeted approach for this rapidly increasing metabolic burden and potentially understand the interplay between MASLD and T2D. This in turn can help to reduce/prevent the future hepatic and cardiovascular complications. While the study by Tinoco *et al*. offers welcome addition to this emerging issue, we call for much more concerted effort to address the gap in evidence and sustainable care pathways for identifying, risk stratification and management of MASLD.

## CRediT authorship contribution statement

AA wrote the 1st draft and PS reviewed and both authors finalised the manuscript.

## Funding

This research did not receive any specific grant from funding agencies in the public, commercial, or not-for-profit sectors.

## Declaration of Competing Interest

Prof Aftab Ala is the associate editor who handled the manuscript by Silva-Tinoco et al and Prof Saravanan is the editor in chief of Clinical Medicine.
